# The effect of plate fixation on supination and pronation of the feline antebrachium: a model of pancarpal arthrodesis

**DOI:** 10.1186/s12917-021-02767-3

**Published:** 2021-01-22

**Authors:** Rachel M. Basa, Matthew J. Allen, Kenneth A. Johnson

**Affiliations:** 1 Sydney School of Veterinary Science, Faculty of Science, University of Sydney, Sydney, NSW Australia; 2 Surgical Discovery Centre, Department of Veterinary Medicine, University of Cambridge, Cambridge, UK

**Keywords:** Arthrodesis, Feline, Pronation, Supination, Plate

## Abstract

**Background:**

Pancarpal arthrodesis is purported to limit supination and pronation of the feline antebrachium. The objective of this study was to investigate whether plate fixation of the radius to the carpus and metacarpus limits supination and pronation of the ulna relative to the radius as a model for pancarpal arthrodesis in the cat. Eight feline cadaveric forelimbs were rotated from supination to pronation in a testing jig and CT (computed tomography) was performed in the neutral, supinated and pronated positions. A locking plate was then secured dorsally to the radius, radial carpal bone and metacarpal III of each of the limbs. CT was repeated in each of the testing positions following plate application. The radius and ulna of the control specimens, and the radius, ulna and plate of the plated specimens were then segmented using software. Alignment of the bones to the radius in the control specimens, and to the plate in the plated specimens was used to compare the changes in degrees of movement of the ulna relative to the radius in dorsal, sagittal and transverse planes.

**Results:**

Based on the results of the paired t test, there was no significant difference in degrees of movement, or total range of motion between control and plated specimens in supinated and pronated testing conditions.

**Conclusion:**

The results of this ex-vivo study indicate that under the testing conditions employed, plate fixation of the radius to the carpus and metacarpus does not limit supination and pronation of the feline antebrachium.

## Background

Feline carpal injuries are commonly caused by high-rise syndrome or motor vehicle accidents and can result in carpal hyperextension when there is damage to the palmar ligaments, flexor retinaculum, or a combination of these structures [1–7]. In the cat, the antebrachiocarpal joint is the most commonly affected carpal joint compartment, followed by the carpometacarpal and middle carpal joints [1–3, 5, 8].

Pancarpal arthrodesis is considered to be a salvage procedure and is commonly performed as a surgical technique in the management of feline carpal injuries [1, 5]. This procedure entails achieving fusion of the carpal joint spaces by adhering to the following principles; articular cartilage debridement, autogenous cancellous bone grafting, and internal fixation with interfragmentary compression and positioning of the carpus at a standing angle [9–11]. The reported indications for pancarpal arthrodesis include hyperextension injury, severe fractures or luxations with chronic mal-alignment, end-stage osteoarthritis and selected neurological deficits [1, 10]. In cases where the antebrachiocarpal joint is unaffected, partial carpal arthrodesis may be recommended [8, 11].

While pancarpal arthrodesis is a commonly implemented surgical treatment for antebrachiocarpal luxation in the dog, its use in cats is more contentious [1]. Cats normally have a greater degree of supination and pronation than dogs – this is considered important for activities such as jumping, climbing and grooming [1, 3–5]. Pancarpal arthrodesis in cats may result in more functional impairment by restricting these activities [3–5]. The largest case series reporting on long-term functional outcome following arthrodesis in cats showed that the height of jump was markedly affected and the ability to jump and climb was mildly affected [5]. Although it is well recognised that pancarpal arthrodesis eliminates normal flexion and extension of the carpus, there are no definitive studies demonstrating that this surgery affects supination and pronation of the feline antebrachium.

Of the various reported techniques for pancarpal arthrodesis, use of a dorsally applied bone plate is the most common [1, 5, 12]. The aim of this study was therefore to use a cadaveric model of pancarpal arthrodesis to describe how the use of a dorsal trans-articular plate affects rotation of the ulna relative to the radius. The null hypothesis was that dorsal plating of the radius to the carpus and metacarpus would limit supination and pronation of the ulna relative to the radius.

## Results

**Fig. 1:**
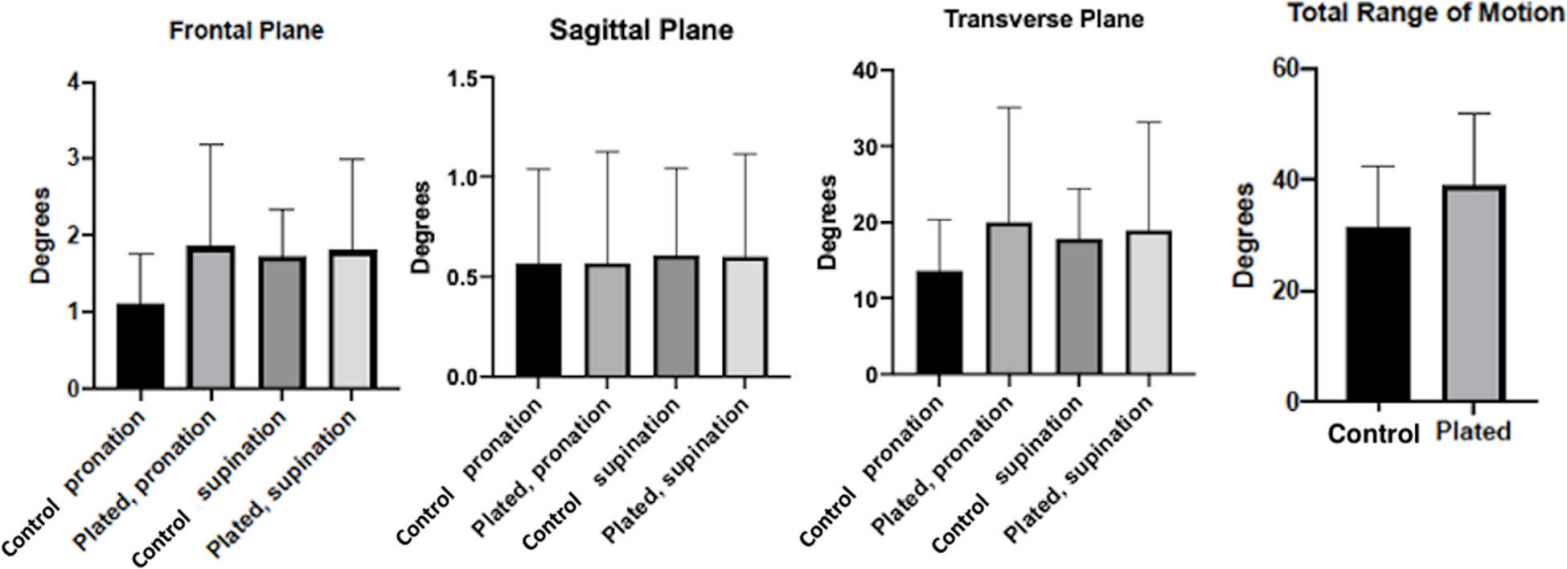
Bar charts illustrating kinematic data obtained from control and plated constructs placed in supination and pronation, in the transverse plane. Data represent mean values for *N* = 8 specimens per test condition. Error bars reflect standard deviation

The results of the paired t test confirmed that there was no significant difference between control and plated specimens in supinated and pronated testing conditions (Table 1, Fig. 1). Similarly, there was no significant difference in total range of motion from pronation to supination in the transverse plane between control and plated specimens (Table 1).

**Table 1:** Relative motion of the ulna and radius (or plate) in transverse, sagittal and frontal planes, and range of motion from pronation to supination in both control and plated specimens. Data are reported in degrees of motion in frontal, sagittal and transverse planes

Specimen I.D.	Control or plated	Pronation			Supination			Range of motion pronation to supination in the transverse plane
		Frontal (abduction/ adduction)	Sagittal (flexion/ extension)	Transverse (pronation/ supination)	Frontal (abduction/ adduction)	Sagittal (flexion/ extension)	Transverse (pronation/ supination)	
Carpus 1	Control	2.12	0.25	15.35	1.48	0.85	17.02	32.37
Carpus 2	Control	2.30	0.62	20.68	0.43	0.92	12.12	32.80
Carpus 3	Control	0.01	0.01	0.29	1.23	0.21	10.06	10.35
Carpus 4	Control	1.04	0.19	11.38	2.12	0.60	21.03	32.41
Carpus 5	Control	0.64	0.31	8.14	1.92	0.27	18.57	26.71
Carpus 6	Control	0.45	0.95	17.60	2.21	0.04	18.93	36.53
Carpus 7	Control	1.14	0.79	16.93	1.56	0.60	13.86	30.79
Carpus 8	Control	1.12	1.42	18.45	2.80	1.37	31.25	49.70
	*Mean*	*1.10*	*0.57*	*13.60*	*1.72*	*0.61*	*17.86*	*31.46*
	*Standard deviation*	*0.78*	*0.47*	*6.72*	*0.72*	*0.44*	*6.56*	*10.89*
Carpus 1	Plated	4.89	0.30	49.05	0.42	0.07	4.21	53.26
Carpus 2	Plated	2.65	1.69	37.75	0.97	0.15	5.79	43.54
Carpus 3	Plated	3.09	1.16	14.61	0.10	0.61	5.26	19.87
Carpus 4	Plated	1.64	0.46	15.23	2.59	0.56	28.42	43.65
Carpus 5	Plated	0.48	0.28	5.66	4.00	1.00	38.61	44.27
Carpus 6	Plated	1.13	0.19	12.97	1.67	0.17	17.95	30.92
Carpus 7	Plated	0.50	0.11	8.85	1.19	0.63	14.07	22.92
Carpus 8	Plated	0.45	0.35	15.97	3.50	1.61	37.26	53.23
	*Mean*	*1.85*	*0.57*	*20.01*	*1.81*	*0.60*	*18.95*	*38.96*
	*Standard deviation*	*1.59*	*0.56*	*15.15*	*1.43*	*0.51*	*14.21*	*12.90*
*Control verses plated paired t tests*		*p = 0.183*	*p > 0.99*	*p = 0.243*	*p = 0.827*	*p = 0.969*	*p = 0.768*	*p = 0.077*

The validity of the measurement methodology assessed by the repeatability study in one set of CT images found that the co-efficient of variation was less than 5%, except for pronation in the sagittal plane, which also had the lowest mean of 0.19 (Table 2).

**Table 2:** Summary of repeatability testing. A complete analysis (model generation plus measurement) was performed five times on a single specimen. Data are reported in degrees of motion in frontal, sagittal and transverse planes. Reproducibility is reported as the coefficient of variation from these repeated measurements

	Pronation			Supination		
	Frontal (abduction/ adduction)	Sagittal (flexion/ extension)	Transverse (pronation/ supination)	Frontal (abduction/ adduction)	Sagittal (flexion/ extension)	Transverse (pronation/ supination)
Original	1.04	0.19	11.38	2.12	0.60	21.03
Repeat 1	1.00	0.18	11.49	2.12	0.60	21.03
Repeat 2	0.99	0.20	11.32	2.07	0.66	20.45
Repeat 3	0.96	0.23	11.27	2.14	0.67	20.30
Repeat 4	1.02	0.15	11.35	2.12	0.65	20.56
*Mean*	*1.00*	*0.19*	*11.36*	*2.11*	*0.64*	*20.67*
*Standard deviation*	*0.03*	*0.03*	*0.08*	*0.03*	*0.03*	*0.34*
*Coefficient of variation %*	*3.03*	*15.43*	*0.72*	*1.23*	*5.29*	*1.63*

Following testing, there was not any evidence of implant failure by plate breakage, screw loosening or breakage, or bone failure such as metacarpal bone fracture.

## Discussion

There was no significant difference between control and plated specimens in supinated and pronated testing conditions, therefore our null hypothesis was rejected (Table 1). This finding suggests that despite the supposition that pancarpal arthrodesis restricts supination and pronation of the feline antebrachium, plate fixation of the radius to the carpus and metacarpus does not limit this motion [4, 5]. Although there were values for supination and pronation, and total range of motion in the plated specimens that were greater than the control specimens, these differences were not significant (Table 1, Fig. 1). The total range of motion was greater in plated specimens 1, 2, 3, 4, 5 and 8 (Table 1). This could be a type II statistical error due to variance or the relatively small sample size.

Cats have a range of pronation (40–50 degrees) and supination (90–128 degrees) that is almost double the values reported in dogs [13, 14]. The mean values for supination (17.86 degrees control, 18.95 degrees plated) and pronation (13.6 degrees control, 20.01 degrees plated) were lower than what has previously been reported in the feline carpus (Table 1). The variability and low values for supination and pronation in the current study may reflect our decision to use moment, rather than degrees of motion (i.e. displacement), to standardise rotation of the antebrachium and would be expected to be much larger in a clinical setting where there is supination and pronation of the entire brachium. This low values for supination and pronation may account for the large range of values for means and standard deviations between specimens (Table 1).

Supination and pronation can be difficult to standardise, and a previous study used a similar methodology to our testing procedure [15]. In that study however, the manus and elbow were fixed at 90 degrees of flexion to replicate Campbell’s test and a protractor was used to measure degrees of supination and pronation [15]. Although standardising degrees of movement may have produced more physiological values for supination and pronation, it theoretically should not affect relative movement between the radius and ulna.

In the present study, aside from the surgical approach and transection of the abductor pollicus longus tendon, the soft tissue attachments of the antebrachium were left intact. The effect of transection of the abductor pollicis longus tendon on carpal range of motion in the cat is unknown. This muscle has its origin on the lateral surface of most of the length of the ulna, and the dorso-lateral surface of the radius, as well as the interosseous ligament. The tendon runs obliquely medial across the carpus to insert on the first metacarpal bone [16]. While the plated limbs, with this muscle transected, had greater degrees of pronation, adduction and total range of motion than the control limbs, these differences were not significant. Furthermore, in using cadavers the effect of weight bearing, and active and passive stabilisers such as the pronator and supinator muscles were also removed [17]. The authors noted that control specimen 3 had the lowest values for total range of motion (10.35 degrees), which potentially could have been the result of metacarpals IV and V slipping in the potted acrylic of the testing jig (Table 1). This may have been avoided by fixing entire manus in acrylic.

This study made use of a titanium 1.3/ 1.7 locking plate, applied as a bridge plate via a dorsal approach. This plate was chosen because titanium is known to create less of a CT (computed tomography) artefact than standard stainless-steel plates [18]. Other implant systems used for feline pancarpal arthrodesis are those that allow for interfragmentary compression and accommodate smaller screws in the distal plate holes, including the Hybrid Pancarpal Arthrodesis (1.5/ 2.0; Veterinary Instrumentation) and CastLess (1.5/2.0; Orthomed) plates [1]. Both of the aforementioned plates are constructed from stainless steel, and therefore would have created greater CT artefact. It has previously been reported that the largest screw size that should be used in feline metacarpal bone III is 1.5 milimetres [6]. Although a medial approach and plating has been reported as a means of performing pancarpal arthrodesis, a dorsal approach and plate were used in this study.

CT was used as a means to assess kinematics of the ulna relative to the radius in this study. The advantage of CT compared to conventional techniques such as radiography and fluoroscopy is that it enables the antebrachium to be studied in three planes (sagittal, transverse and frontal). Based on the results of this study, it was observed that there is minimal movement of the ulna in the frontal and sagittal planes during supination and pronation of the antebrachium (Table 1, Fig. 1).

Kinematics were assessed by using a computer-based technique to generate three-dimensional surface models that were aligned to a fixed object (either the radius in control specimens, or the plate in plated specimens). The repeatability of this technique was validated by repeating the analysis five times on a control specimen and was confirmed to be within an acceptable range (Table 2). It could be argued that the same reference object should have been used for the control and plated specimens, however the plate was considered to be a surrogate for the radius given that it was fixed to the bone. The coefficient of variations generated based on the repeatability testing confirm that most values were less than 5% except for pronation in the sagittal plane (Table 2). This is likely the result of the mean being so small in this plane and direction, therefore creating a narrower margin for error.

As with any ex-vivo model, this study was not without limitations. Firstly, the principles of arthrodesis were not adhered to. In a clinical setting the surgeon would perform a surgical approach, incise the joint capsule and burr away articular cartilage, then insert autogenous cancellous bone graft within the joint spaces before applying internal fixation to stabilise the carpus at a standing angle. Secondly, there is no opportunity for the arthrodesis to heal in a cadaveric study, so any limitations of motion reflect changes due to the plate alone, not the fusion that occurs across the carpal bones. Although there are studies reporting osseous fusion times in the dog, there is no such information in the cat and the reports of feline clinical cases anecdotally refer to fusion times of 3–4 months [8, 12, 19, 20].

Aside from there being lack of bone activity, the current study does not fully emulate an arthrodesis if it were assumed that there is eventual fusion of the ulnocarpal joint. The distal ulna is more bulbous in the cat than the dog, and therefore has a small articulation with the proximal row of carpal bones [21]. Based on our results, there may be an argument to preserve the ulnocarpal joint in the cat. A future area of research would be to prospectively evaluate supination and pronation in clinical patients both pre- and post-pancarpal arthrodesis at various time points to assess whether progressive osseous fusion of the entire antebrachiocarpal joint affects antebrachial rotation.

## Conclusion

In conclusion, the results of this ex-vivo study indicate that plate fixation of the radius to the carpus and metacarpus does not limit supination and pronation of the feline antebrachium.

## Methods

### Specimens

Eighteen thoracic limbs were obtained from nine domestic short- haired cats weighing 2.9–4.9 kg that were euthanized for reasons unrelated to the study and acquired in accordance with guideline GL001 from the University of Sydney animal ethics committee. The limbs were amputated at the level of the mid humerus and each limb was radiographed with orthogonal views of the carpus and elbow to ensure that they were free of skeletal disease. Cats were excluded from the study if they were less than 266 days old, as determined by the olecranon physis of the ulna being open [22]. This was the case in one set of limbs, which was excluded from the study. A random number generator was used to determine which sided limb was used from each specimen, with one limb being selected from each cat. Each thoracic limb was wrapped in surgical gauze soaked with saline solution stored at -20C.

### Description of experimental equipment

A custom- made jig was used for testing each limb in supinated and pronated positions. The construct was made from wooden blocks (dimension 4x30x15cm) arranged to create a platform that allowed for a carbon rod to suspend each antebrachium at the proximal aspect of the jig.

In brief, the proximal aspect of the jig consisted of two parallel blocks on either side separated by a gap, which served as a platform for placement of the limb. The limbs were suspended by using a carbon rod inserted in a medial to lateral direction across the lateral and medial parts of the humeral condyle. The distal aspect of the jig contained a hole that allowed for insertion of an extension drive which was attached to a socket adaptor with a universal joint. This allowed for the attachment of a stainless-steel socket. A male adaptor was inserted at the distal end of the extension drive, which allowed attachment of a torque- limiting screwdriver during CT scans. The limb was secured in supinated and pronated positions with a 250 N.mm moment in each direction during image acquisition.

### Specimen preparation and mounting

Each limb was kept at room temperature for 12 h prior to testing and soft tissues distal to the mid humerus were left intact. The digits were amputated at the level of the metacarpophalangeal joint. The limb was suspended in the testing jig and the manus (at the level of distal metacarpals III and IV) was potted in acrylic within the previously described socket. Alignment of limbs in the jig was standardised by ensuring that the olecranon tuber was orientated perpendicular to the base of the jig, and the angle of the carpus was 180 degrees. The soft tissues of the elbow were left intact and there was unconstrained movement of the elbow. Further description of the experimental equipment, specimen preparation and mounting are provided in a previous publication [17].

### Specimen testing and CT acquisition

The jig containing the cadaveric limb was positioned in a CT scanner (16 slice Philips Brilliance helical CT, Amsterdam, Netherlands) and scanned with the following scan parameters: 120KV, 200mAs, slice thickness 0.8 mm and ultra- high resolution. Each limb was imaged from the distal humerus to the metacarpals in a neutral position and then with a 250 Newton millimetre (N.mm) supinating moment, followed by a 250 N.mm pronating moment.

### Surgical approach to the carpus

**Fig. 2:**
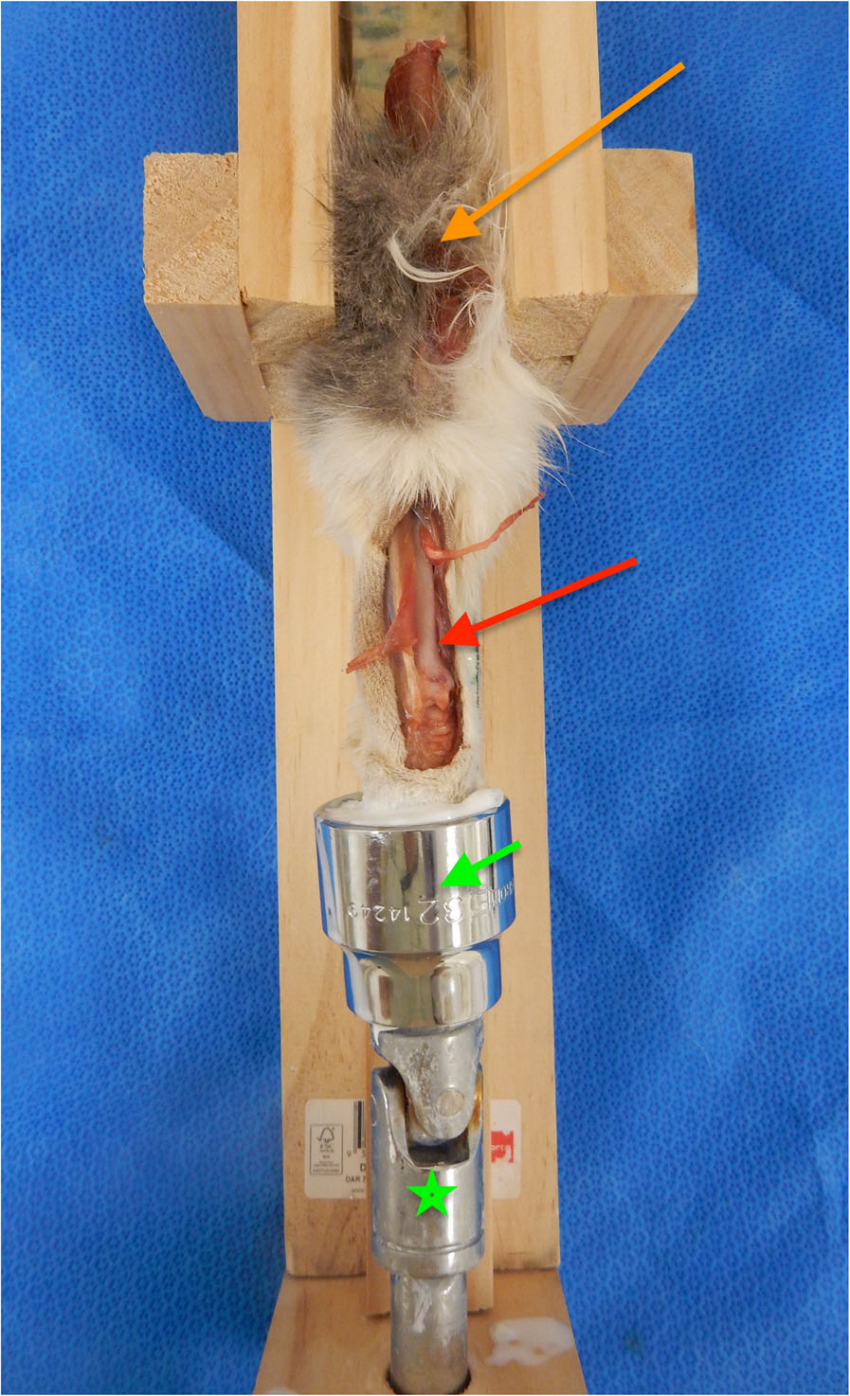
Frontal view of the testing jig with specimen. A dorsal approach to the carpus has been performed in this specimen. The orange arrow identifies the location of a carbon fibre rod that is inserted within the distal humerus to suspend the antebrachium. The red arrow shows where a dorsal approach to the distal radius and carpus has been made. The green arrow shows the location of the socket in which metacarpals III and IV and potted in acrylic, and the green star identifies extension drive

A standard dorsal approach was made to the distal radius and carpus with the antebrachium remaining fixed within the testing jig (Fig. 2) [23]. The abductor pollicus longus tendon was transected at its insertion to allow for accurate plate application to the radius, and the joint capsule was elevated from the proximal carpal row and distal radius to enable better visibility of the dorsal surface of the radial carpal bone.

### Plate application

**Fig. 3:**
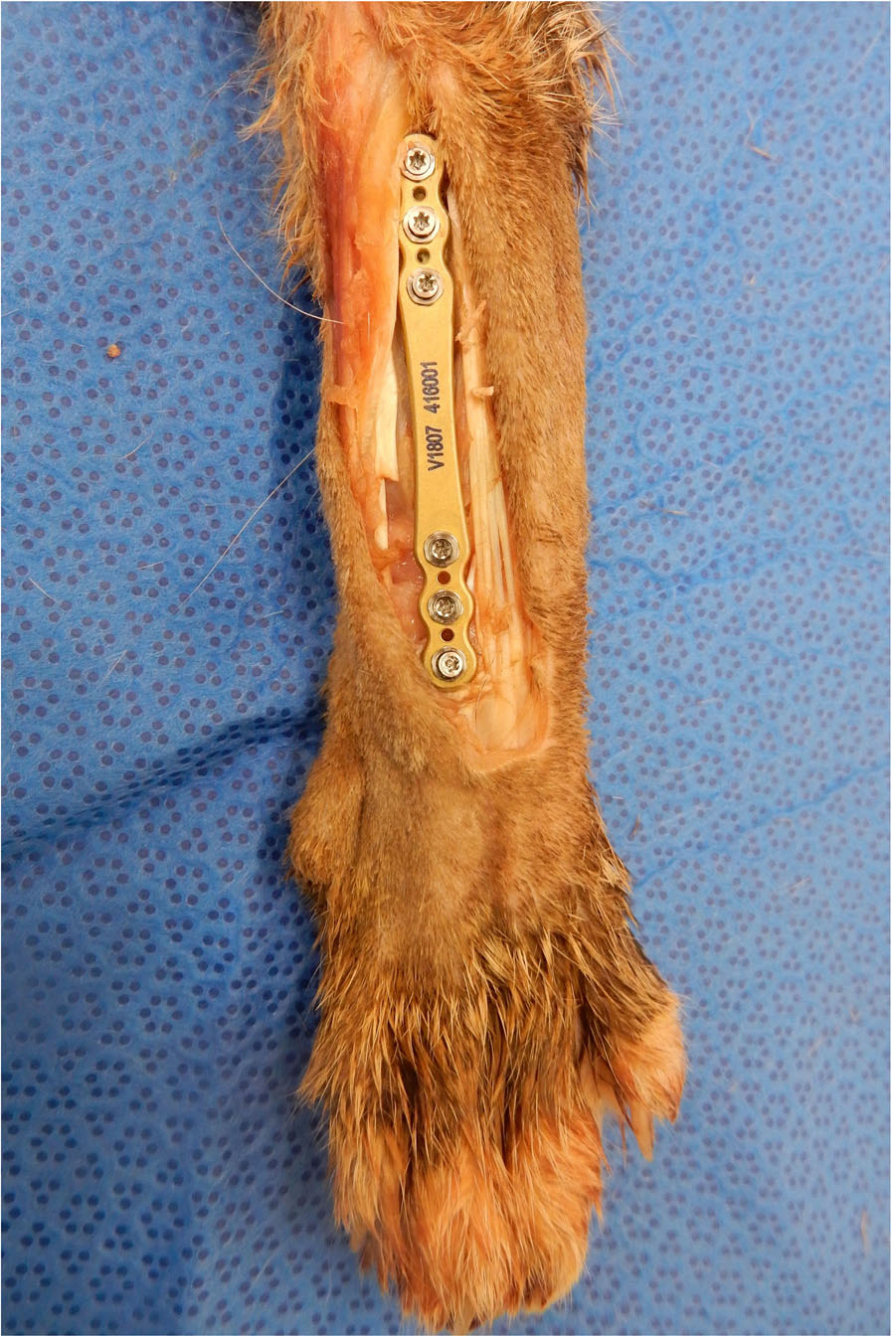
Image for demonstration of a test specimen without placement in the testing jig. Dorsal approach to the distal radius and carpus has been made, and a six hole, 1.7 mm conical coupling locking plate is secured to the bone with the three most proximal screws engaging the radius, the fourth screw engaging the radial carpal bone and the two most distal screws engaging metacarpal III

A six hole, 1.7 mm conical coupling titanium alloy locking plate, 47 mm long and 1.2 mm thick (Fixin 1807 InTrauma micro series) was applied to each carpus (Fig. 3). Bone holding forceps were used to secure the plate to the bone. The fourth most distal hole was firstly drilled with a 1.3 mm drill bit and a 1.7 screw was inserted into the radial carpal bone. The three most proximal holes were then drilled and 1.7 mm screws inserted, followed by the two remaining distal holes which engaged the third metacarpal bone. The screws varied in length from 6 to 10 mm.

### CT acquisition of the plated specimens

After the plate was applied to the carpus of each specimen, CT acquisition was repeated with the same parameters used for the first scans. The ‘scout’ scans were examined to confirm appropriate arthrodesis implant application and positioning, before proceeding with the second set of CT scans in the neutral, supinated and pronated positions. There was a total of 6 CT scans for each of the limbs (3 in neutral, supinated and pronated positions of the control specimens; 3 in neutral, supinated and pronated positions of the plated specimens). Following the scout scan in 3 of the limbs, the fourth most proximal screw did not engage the radial carpal bone. In those cases, either the screw was re-inserted, or the plate was repositioned on the bone until implant position was deemed to be adequate.

### Image segmentation

Computed tomography data were stored in a Digital Imaging and Communication in Medicine (DICOM) format for later processing. DICOM images were imported into a DICOM image processing software (Materialise Mimics vs 19.0, Leuven Belgium). The radius, ulna and plate were segmented and three-dimensional (3D) iso-surface models were created. The 3D bone models were saved as .stl (surface tessellation language/ stereolithography) files types for subsequent analysis.

### Creation of frontal, sagittal and transverse planes and calculating angles

**Fig. 4:**
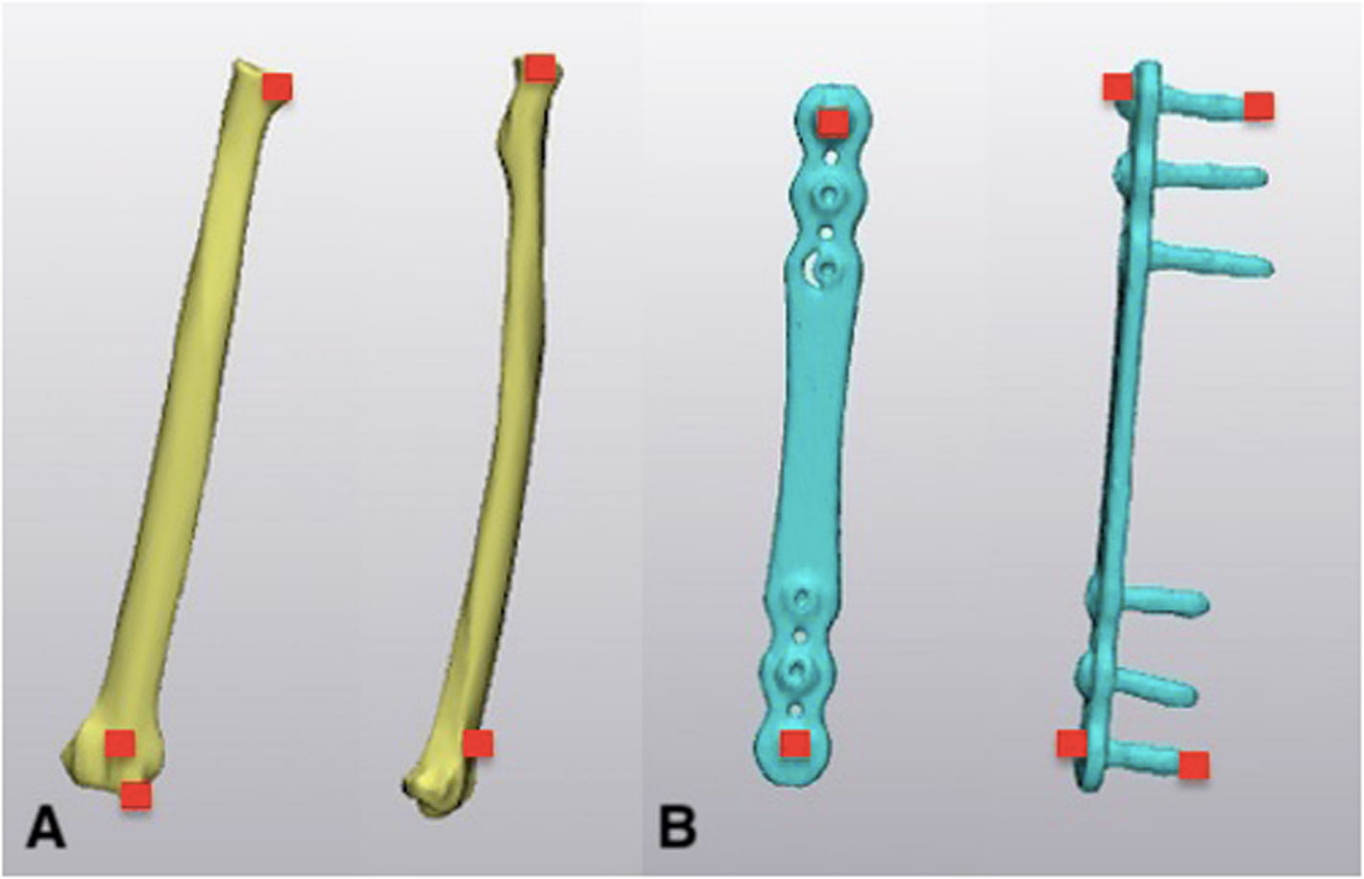
Fiducial points that were to align the radius (**a**) in the control specimens and the plate (**b**) in the plated specimens are marked as red rectangles on the diagrams. On the radius, the landmarks included the most medial and lateral aspects of the radial head, with a distal marker used on the radial styloid and most proximal aspect of the dorsal tubercle. On the plate, the points used were the heads and tips of the first and last screws

**Fig. 5:**
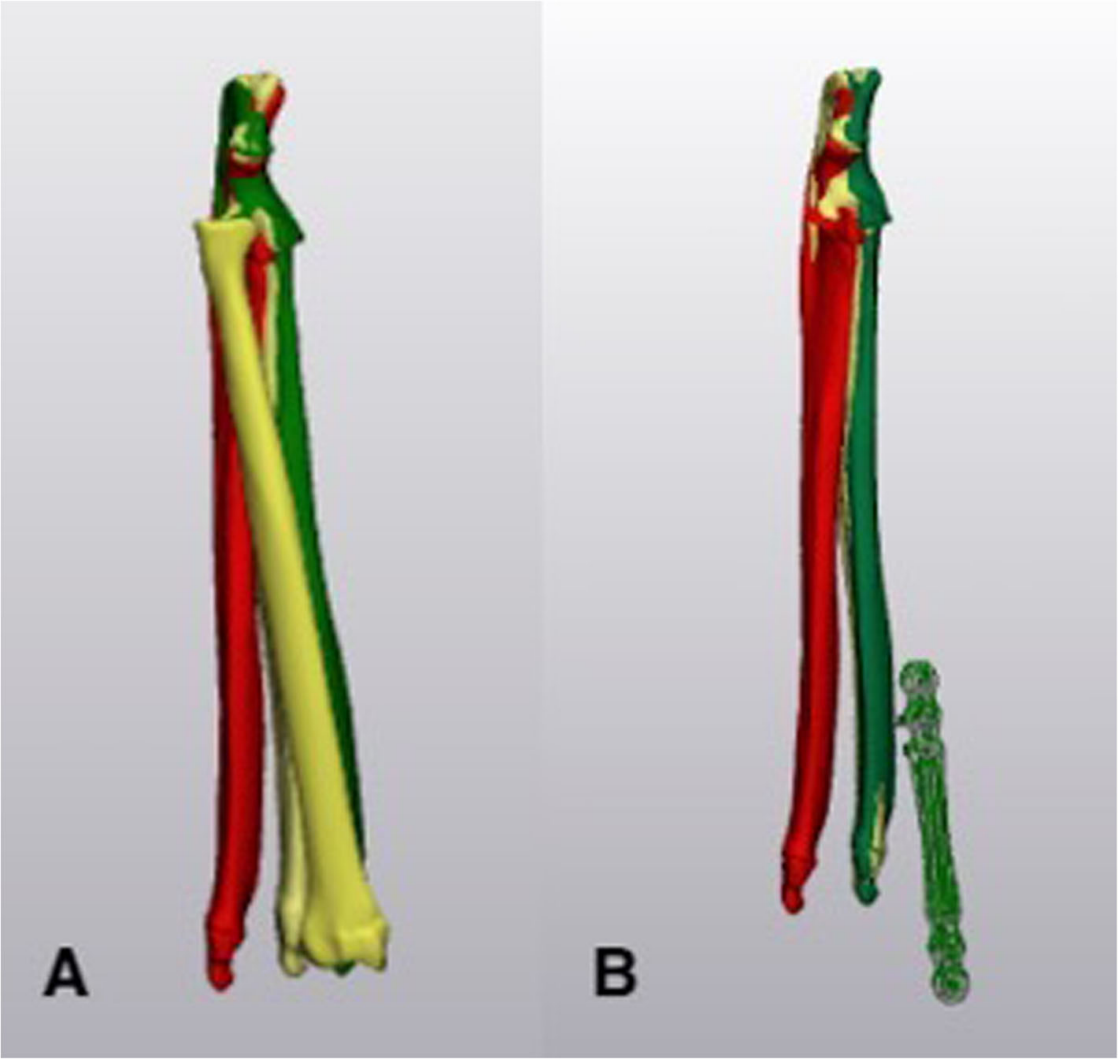
The radius was used as the point of reference in the control specimens (**a**), and the plate in the plated specimens (**b**). This allowed for relative movement of the ulna to be measured between the neutral, supinated and pronated positions. In the images, the yellow ulna is in the neutral position; the green ulna in the pronated position and the red ulna in the supinated position

The .stl files for each specimen were imported into a software program that allowed for alignment to be assessed in the different testing conditions (Materialise 3Matic, vs 11.0, Leuven, Belgium). In the control specimens, the neutral radius was used as the point of reference for motion of the ulna. In the neutral plated specimens, the plate was used as the point of reference because the presence of the bone plate obscured the dorsal surface of the radius. Multiple fiducial points were identified on the radius and the plate and used as registration points for alignment of the images from the neutral, supinated and pronated scans (Fig. 4) [24]. After point registration, 2–3 cycles of global registration were used to ensure that there was complete alignment of the radii or plates for each specimen. The relative motion of the ulna in supination and pronation was determined using the neutral scan as the reference (Fig. 5). Measurements were made in three planes – frontal (varus/valgus), transverse (pronation/supination) and sagittal (flexion/extension) – and reported in degrees.

### Validation of methods: assessing for repeatability

The CT scans of one specimen (specimen 5 control), positioned in neutral, supinated and pronated positions were separately thresholded five times. The radius and ulna bone models were imported into software (Materialise 3Matic, vs 11.0, Leuven, Belgium) and the relative movement of the ulna determined as described above. Means, standard deviations and coefficient of variation were calculated from these data.

### Statistical analysis

Data were imported into a commercial statistical analysis package (Prism version 8.0 for Mac; GraphPad Software, LLC) and the results for paired control (non-plated) and plated specimens were compared using a paired Student’s t-test in both supinated and pronated positions, with significance set at *p* < 0.05 (Table 1).

## Data Availability

The datasets used and/or analysed during the current study are available from the corresponding author on reasonable request.

## Abbreviations

CT: Computed tomography
